# Expression analysis of inflammasome sensors and implication of NLRP12 inflammasome in prostate cancer

**DOI:** 10.1038/s41598-017-04286-4

**Published:** 2017-06-29

**Authors:** Dev Karan, Ossama Tawfik, Seema Dubey

**Affiliations:** 1 Department of Pathology, Microbiology and Immunology, University of South Carolina School of Medicine, Columbia, SC USA; 2 Department of Pathology and Laboratory Medicine, University of Kansas Medical Center, Kansas City, KS USA

## Abstract

Inflammasomes are multi-proteins complex regulating inflammation-associated signaling. While inflammation plays a critical role in cancer cell growth, studies remain uncharacterized on the role of inflammasomes in prostate cancer. Using Gene Expression Omnibus (GEO) public datasets, we screened the expression profiles of inflammasome sensors NLRP3, NLRC4, NLRP6, NRLP12, and AIM2 in prostate tumor tissues, and verified their mRNA level in a panel of prostate cancer cell lines. The selected expression of NLRP3 and NLRP12 inflammasomes was validated, and the clinical association was evaluated in human prostate archival tumor tissues. We observed that the expression of inflammasome sensors was dysregulated at the mRNA level except for the NLRP12. The intensity of NLRP12 immunostaining was significantly higher in malignant prostate as compared to their adjacent benign tissues. In contrast, the NLRP3 immunostaining in prostate tissues was heterogeneous. The inflammasome complex proteins ASC (apoptosis-associated speck-like protein containing a CARD) and pro-caspase-1, as well as its downstream targets IL-1β and IL-18 were confined to aggressive prostate cancer cells. These data suggest an increased expression of NLRP12 in association with prostate cancer and support the role of NLRP12 inflammasome complex regulating inflammatory cytokines in understanding the role of inflammation in the prostate cancer.

## Introduction

Chronic inflammation has long been associated with various types of cancers with an infectious etiology. Specifically, the impact of inflammation in prostate cancer is well-accepted. Multiple studies have demonstrated that prostate cancer cells secrete a variety of inflammatory cytokines mediating tumor cell growth and aggressiveness^1–3^. The role of IL-6 and IL-1β is implicated in bone metastasis of prostate cancer cells^4–6^. Indeed, the inflammatory cytokines fuel the growing tumor microenvironment and provoke tumor cells in achieving their goal of invasiveness and metastasis. However, the molecular basis of regulating inflammation remains largely unknown.

Recent studies have highlighted the role of inflammasomes as a strategic protein complex, providing a molecular platform initiating signaling cascades of inflammatory events. Most of the inflammasomes contains NOD-like receptor (NLR) sensor molecules that includes NLRP1 (NOD-, LRR (leucine rich repeat)- and pyrin domain-containing 1), NLRP3, NLRP6, NLRP7, NLRP12 or NLRC4^7^. Another inflammasome complex of the pyrin and HIN domain-containing protein family member contains AIM2 (absent in melanoma 2) inflammasome sensor^8^. In addition to a receptor, the inflammasome complex consists of ASC (apoptosis-associated speck-like protein containing a carboxyterminal CARD) and pro-caspase-1^9, 10^. The inflammasome sensors can detect a wide variety of danger signals such as pathogen associated molecular patterns (PAMPs) or damage associated molecular patterns (DAMPs)^11^. This process leads to assembly of NLR or AIM2 associated inflammasomes complex, activating caspase-1 to cleave interleukins (IL)-1β and IL-18 into their mature forms stimulating multiple biological effects associated with infection, inflammation and other disease processes^10–12^.

The prostate tissue is a prime target for inflammation secondary to infection caused by various types of microorganisms (e.g. bacteria), or injury by uric acid crystals, serving as danger signals regulating inflammatory processes. In recognition of danger signals, toll like receptors (TLRs) are the most studied receptors, and it’s activation in prostate cancer is associated with the stimulation of various cytokines including IL-6 and IL-1β^13, 14^. As a matter of fact, injury or infection in the prostate is a common event that may trigger inflammasome assembly potentially driving inflammation modulating prostate pathobiology. A diabetic rat model study showed that reducing uric acid level in serum prevented NLRP3 inflammasome activation and helped improving renal function^15^. Various inflammasomes are responsible for activation of inflammatory processes, and have been extensively characterized in the cells of myeloid origin such as monocytes and macrophages. In this study, we analyzed the expression of various inflammasome sensors in association with prostate cancer. We observed that constitutive expression of NLRP12 inflammasome sensor is significantly higher in prostate cancer as compared to adjacent benign tissues. The dominant expression of inflammasome complex proteins (ASC and pro-caspase-1) in aggressive prostate cancer cell lines implicate the role of NLRP12 inflammasome provoking inflammatory events via regulating IL-1β and IL-18 in the development and progression of prostate cancer.

## Results

### GEO profiles on inflammasome sensors expression in prostate tumors

**Figure 1:**
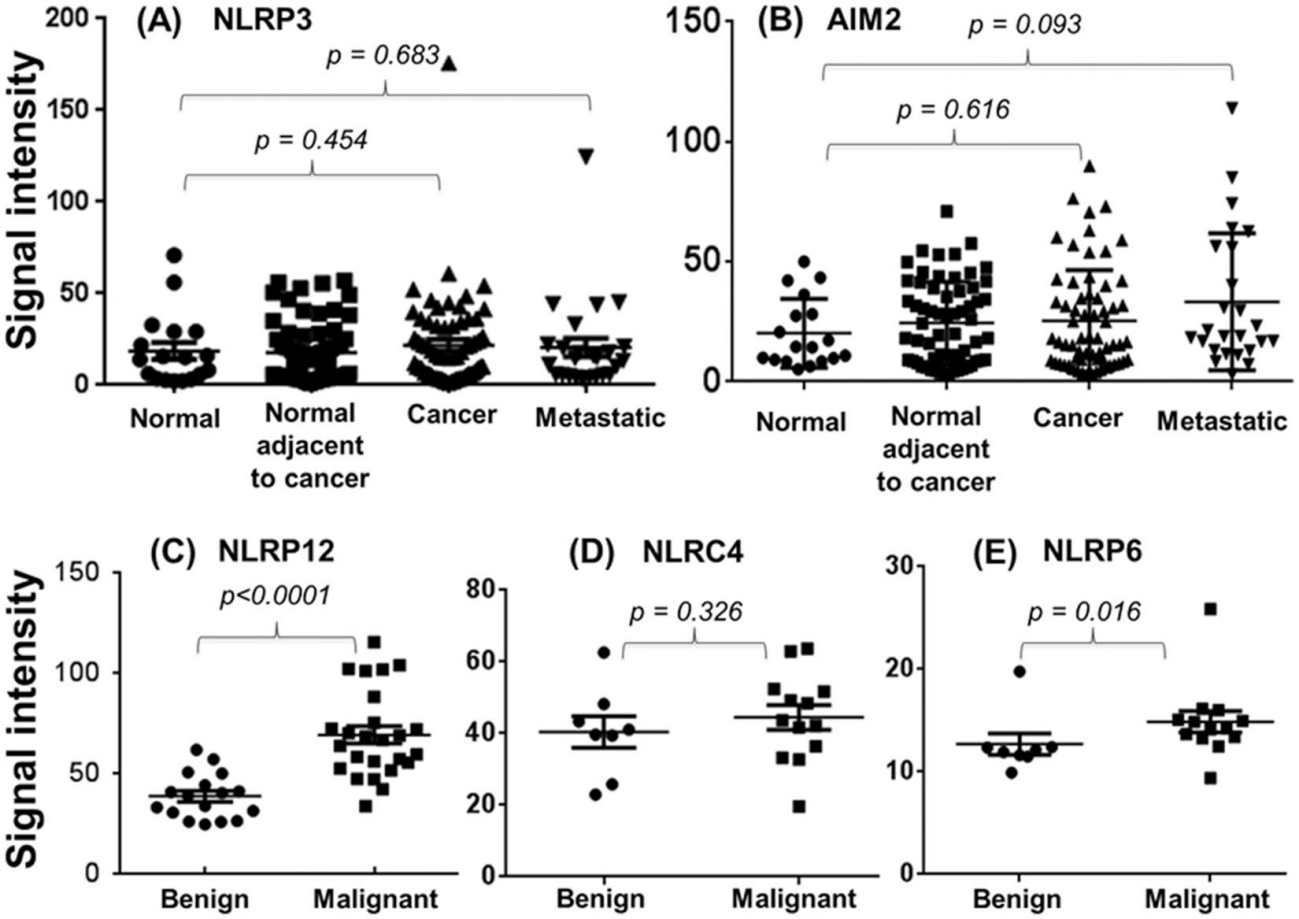
Analysis of gene expression based on microarray datasets from the GEO profiles. The raw values were obtained from the datasets representing a maximum number of prostate samples. Unpaired, non-parametric, Mann-Whitney test was used to analyze the significant level of (**A**) NLRP3; (**B**) AIM2; (**C**) NLRP12; (**D**) NLRC4; and (**E**) NLRP6 expression between benign and malignant prostate tissue samples. The values are expressed as the standard error of the mean (m ± SE).

To understand the role of inflammasomes in prostate cancer, we screened the expression profiles of various inflammasome sensors in prostate tumors examining multiple datasets from the Gene Expression Omnibus (GEO) profiles. We selected a dataset with maximum number of prostate tumor samples. GEO profile dataset (GDS2545) of 171 prostate tissues showed no significant differences between normal vs cancer tissues for NLRP3 (p = 0.454) and AIM2 (p = 0.616), and the expression levels in normal (no pathological alterations in the prostate tissues; n = 18), normal adjacent of tumor (n = 63), tumor tissues (n = 65), and metastatic prostate cancer to various anatomical sites (n = 25) for both the genes were almost similar (Fig. 1A and B). In this dataset of 171 prostate tissues, NLRP12 gene profile was not available, therefore, we pooled two references of the dataset (GDS4824) with similar values, and observed that NLRP12 expression was significantly higher (p < 0.0001) in malignant tissues as compared to adjacent benign tissues (Fig. 1C). Further analysis of the GDS4824 dataset revealed similar expression for NLRC4 (p = 0.326) between adjacent benign and malignant tissues (Fig. 1D), while NLRP6 expression was marginally significant (p = 0.016; Fig. 1E).

### Dysregulated expression of inflammasome sensors in prostate cancer cells

**Figure 2:**
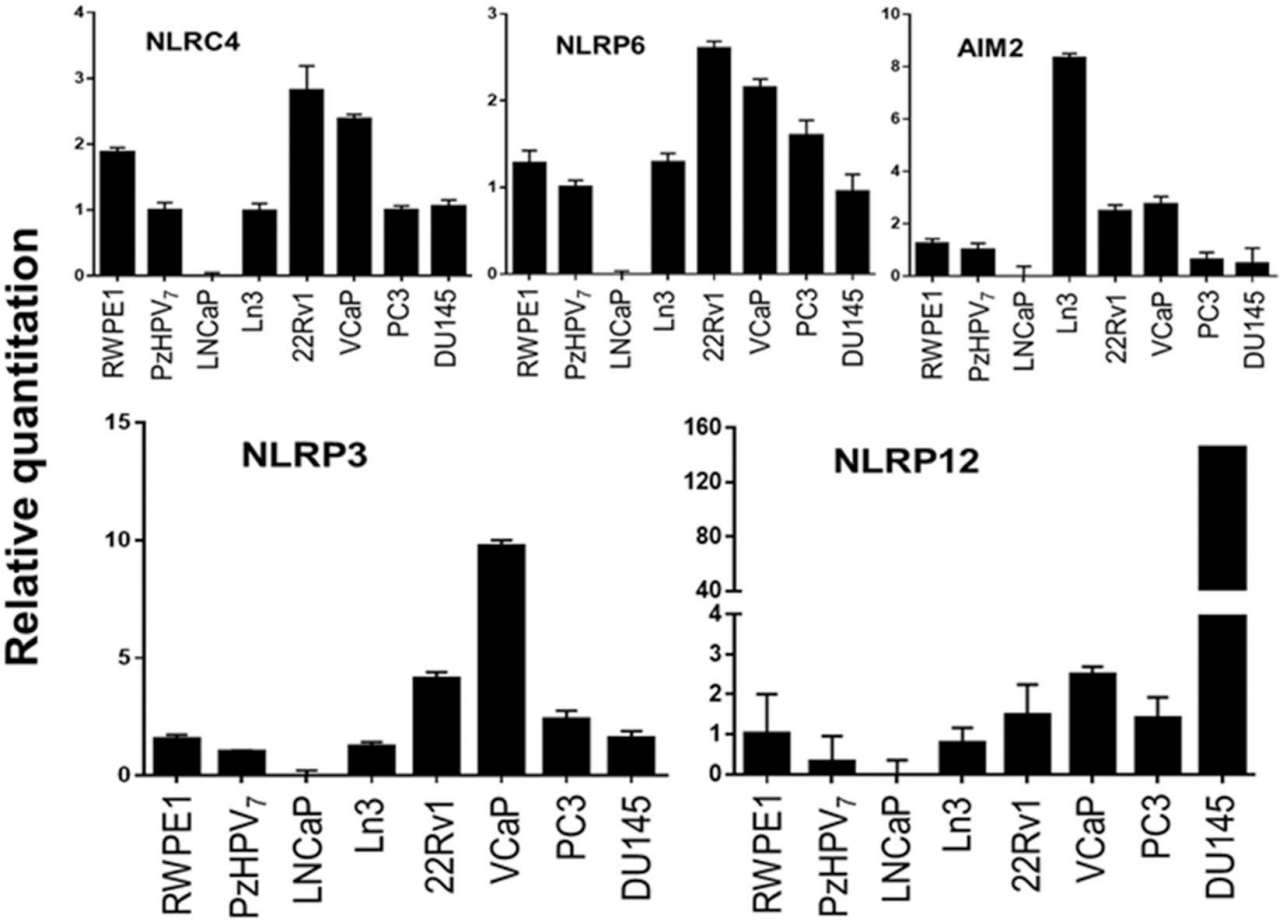
The mRNA expression levels of various NLR receptors (AIM2, NLRP3, NLRC4, NLRC6, and NLRP12) of the inflammasome complex in a panel of human prostate cancer cell lines. The mRNA level was analyzed using quantitative PCR using GAPDH as internal control. The experiment was repeated multiple times from different cell preparations.

To experimentally support the GEO expression profiles of inflammasome sensors, the qPCR analysis showed a similar dysregulated expression of NLRP3, NLRC4, NLRP6, and AIM2 in a panel of human prostate cancer cell lines (Fig. 2). We found that the mRNA level was very much variable across the cell lines, and there was no consensus on the expression levels of NLRP3, NLRC4, NLRP6, and AIM2 among early stage (e.g. LNCaP and LNCaP-Ln3) and late stage (e.g. PC3 and DU145) prostate cancer cell lines. However, mRNA level of NRLP12 was very high in aggressive DU145 cells as compared to other tested prostate cancer cell lines (Fig. 2).

### Expression of NLRP3 and NLRP12 in archival specimens of human prostate tissues

**Figure 3:**
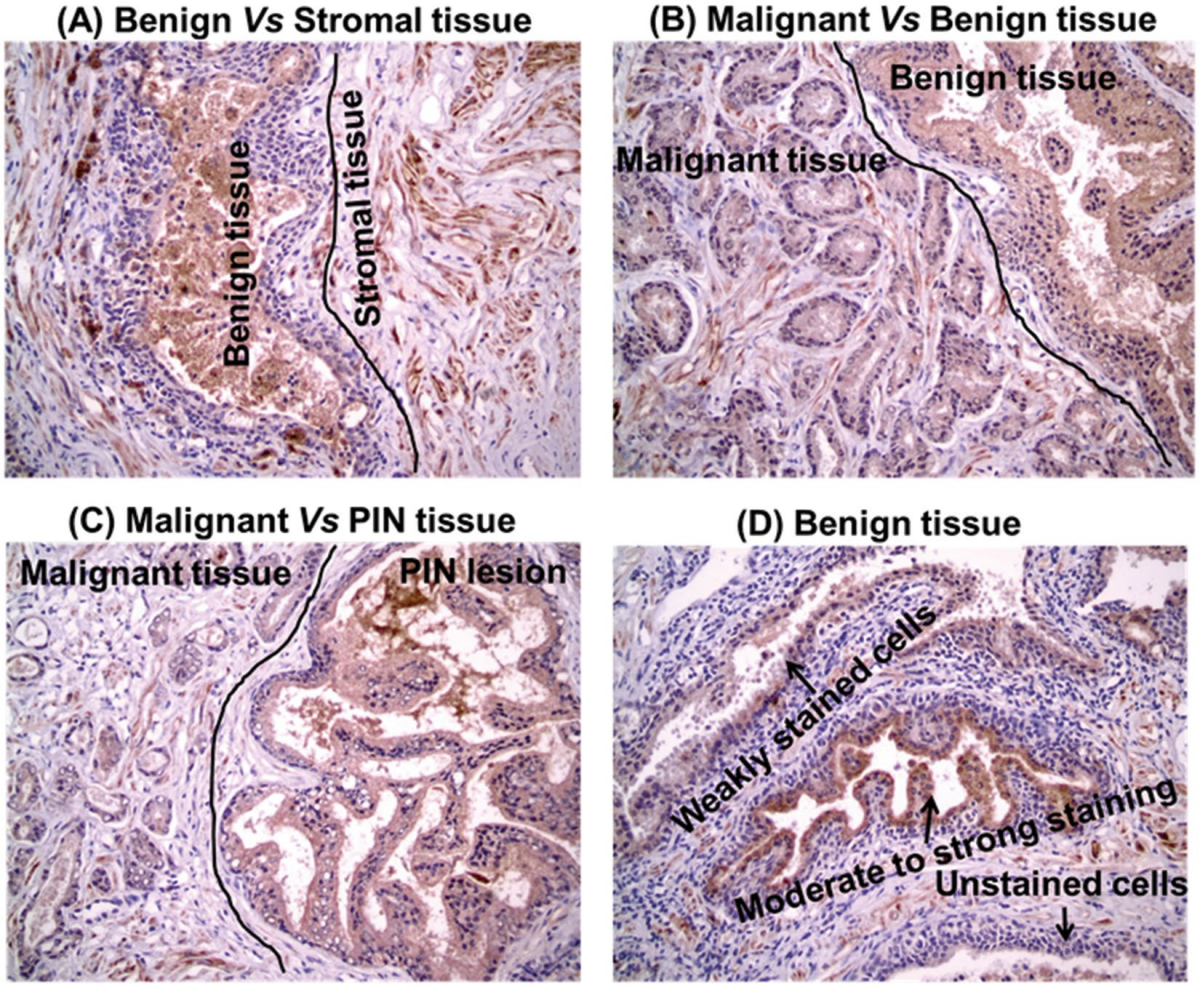
Representative analysis of immunostaining for NLRP3 expression in human prostate archival tissue specimens (n = 19). (**A**) moderate to strong staining in stromal tissues as compared to benign tissue areas; (**B**,**C**) homogeneous staining among malignant vs benign (**B**) and malignant vs PIN lesions (**C**); and (**D**) heterogeneous expression of NLRP3 protein in benign tissue area showing unstained, weakly stained, and moderate to strongly stained cells as marked by the arrow signs.

We further analyzed the clinical relevance of NLRP3 and NLRP12 in archival human prostate tumor specimens using IHC. The comparative analysis between adjacent benign, intraepithelial neoplasia (PIN) and malignant tissues for NLRP3 or NLRP12 is from the same slide of cancerous tissue. Since this is the first study analyzing the expression of NLRP3 and NLRP12 in human prostate tissues, we examined the details of staining pattern in epithelial and stromal tissues. The NLRP3 protein staining from the 19 human prostate tissues revealed that in majority of the cases, 95% of the stromal cells (ranged from 50 to 100%) were NLRP3 positive with moderate to strong staining (Fig. 3A). The adjacent benign, PIN, and malignant tissues showed >95% of cells (ranged from 60 to 100%) positive with weak to moderate staining for NLRP3 (Fig. 3B and C). No significant differences in the intensity of NLRP3 staining were observed between adjacent benign and malignant tissues (p = 0.526). However, the NLRP3 staining between stromal vs. benign epithelial cells (p = 0.004), and stromal vs. cancer cells (p = 0.0006) appeared significantly different (Fig. 3). Additionally, no significant correlation between NLRP3 intensity in stromal tissue vs. PSA (r = −0.25) or cancer vs. PSA was observed. Overall, the NLRP3 staining was very heterogeneous in prostate tissues (Fig. 3D). Since there were no observed differences in NLRP3 expression between the adjacent benign and malignant tissues, we did not extend the analysis of NLRP3 in additional archival prostate tissues.

**Figure 4:**
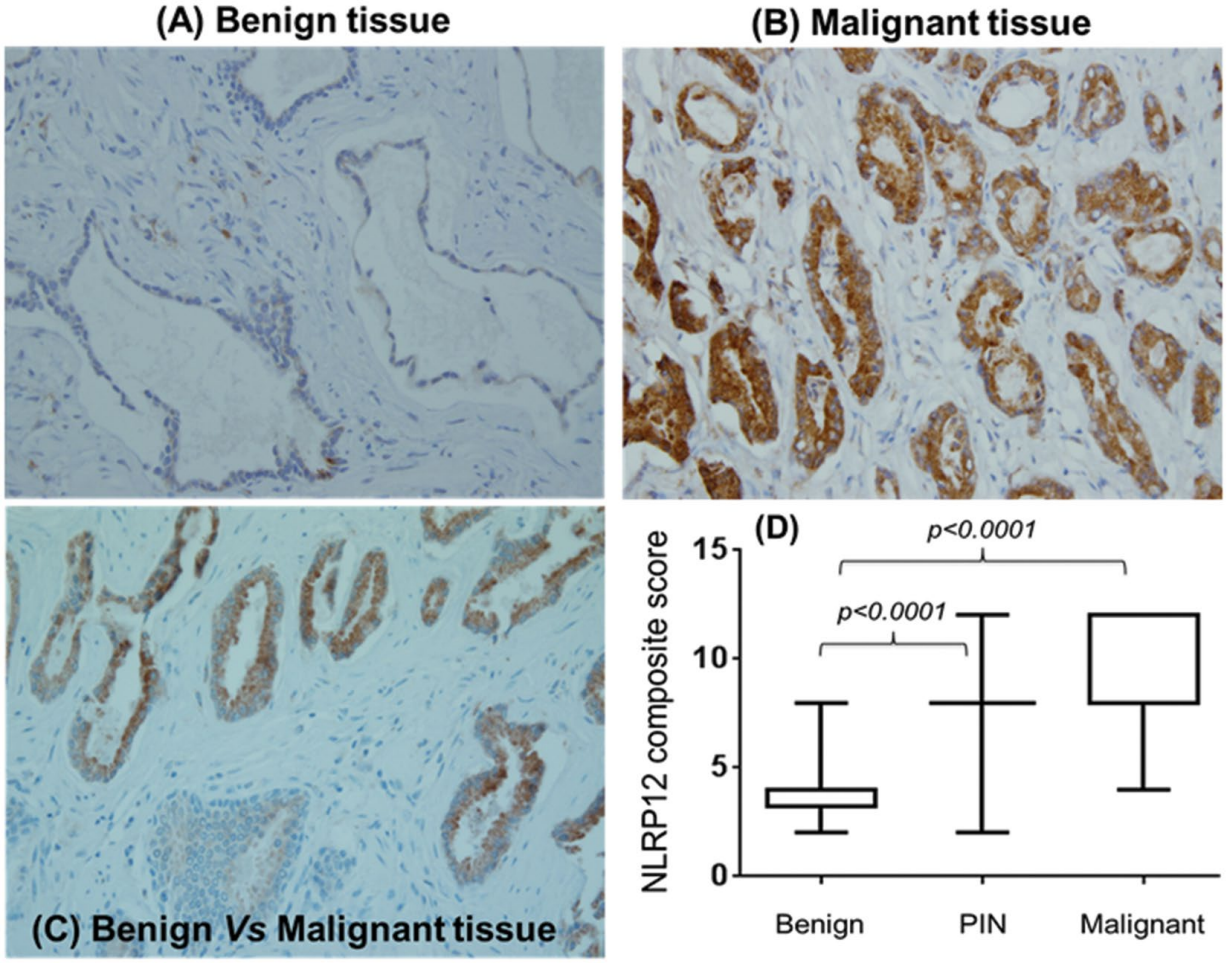
Representative immuno-stained slides for NLRP12 protein expression in human prostate archival tissue specimens (n = 40). (**A**) benign tissue, (**B**) malignant tissue; (**C**) immunostaining distribution between benign and malignant tissue in the matched sample; and (**D**) overall composite score in BPH, PIN, and malignant tissues with a significant increase (p < 0.0001) of NRLP12 protein in malignant and PIN as compared to benign tissue. The values are expressed as the standard error of the mean (m ± SE).

For NLRP12, we performed IHC on 50 archival specimens of human prostate tumor tissues. Of these, 10 specimens did not have either adjacent benign, PIN, or malignant tissues, therefore, not included in the analysis. In contrast to the NLRP3 expression, we found that NLRP12 protein is significantly higher in human prostate tumor tissues as compared to adjacent benign tissues (Fig. 4). The mean value of the composite score in the adjacent benign tissue was 4.35 ± 0.278 (mean ± SE) while the intensity of staining on the same slide in the matched malign tissue was 10.10 ± 0.404. In all of the analyzed specimens (n = 40), the expression of NLRP12 protein was very uniform, cytoplasmic, and specifically restricted to epithelial cells. No staining for NLRP12 protein was observes in stromal cells. Almost 100% of the cells in prostate tissues showed very low to non-detectable NLRP12 protein expression in adjacent benign tissues, and strong NLRP12 expression in PIN and malignant tissues (Fig. 4). Further correlative analysis did not reveal clinical association of NLRP12 with age (−0.043), PSA (−0.122), or Gleason’s score (GS: −0.264). This could be largely due to the fact that the sample size is too small, and the majority of these samples belong to GS 7 (24/40), while 11 samples for GS 6, and 5 samples combined for GS 8 & 9. There was no difference in NLRP12 intensity score in malignant tissues when compared between GS 6 (10.18 ± 0.63) and GS 7 (10.67 ± 0.52). However, overall protein expression level of NLRP12 differ significantly (p < 0.0001) between the adjacent benign and malignant prostate tissues (Fig. 4D).

### Analysis of inflammasome complex proteins in prostate cancer cell lines

**Figure 5:**
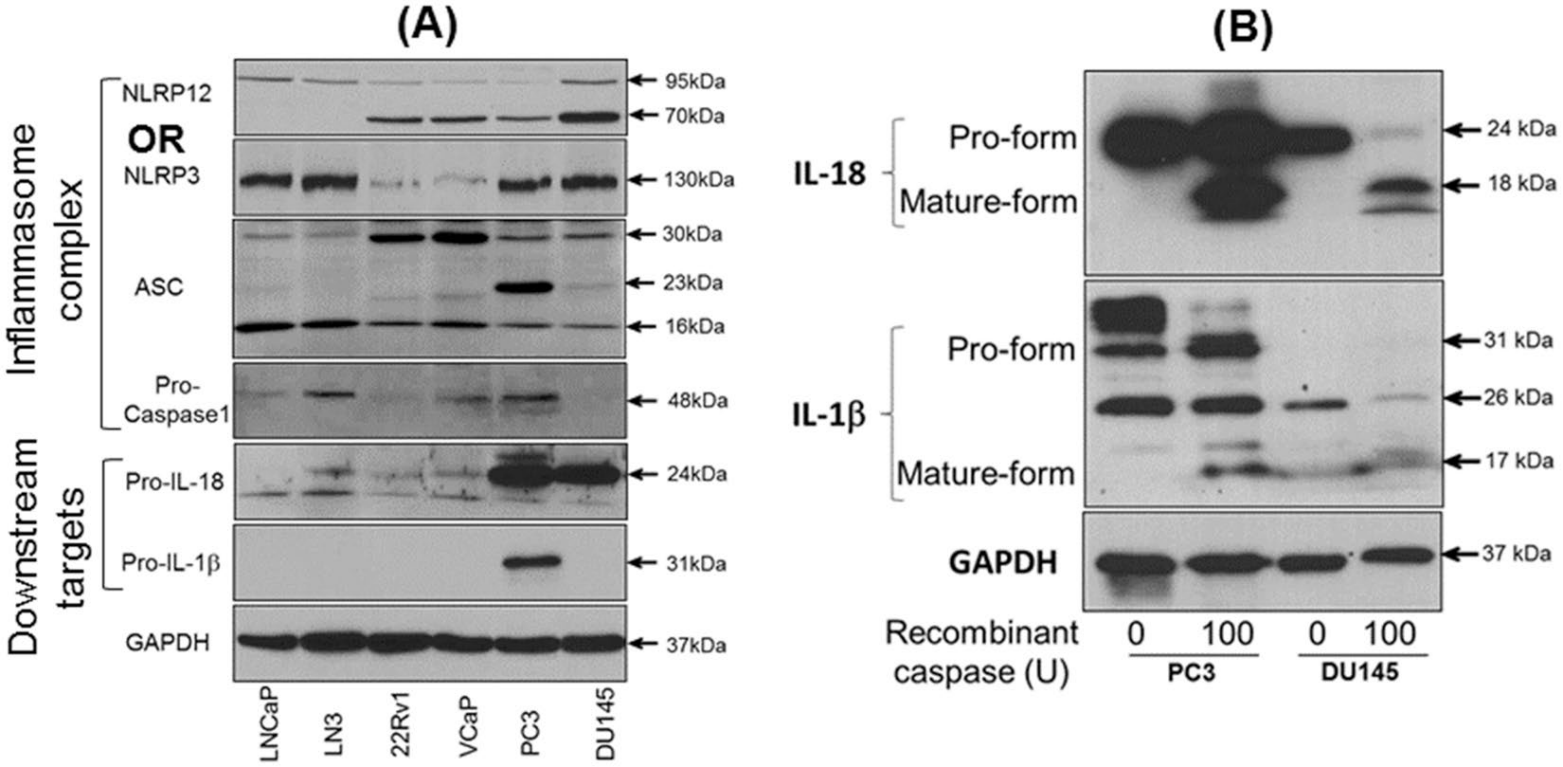
Protein analysis of (**A**) NLRP3, NLRP12, ASC, and pro-caspase-1 inflammasome complex proteins and its downstream targets IL-1β and IL-18 in human prostate cancer cell lines. GAPDH is a representative blot. (**B**) Cleaved and mature forms of IL-1β and IL-18 following digestion of protein cell lysates from PC3 and DU145 cells with human recombinant caspase-1. GAPDH serves as internal control.

To further understand the role of inflammasomes in prostate cancer, we analyzed the protein expression of inflammasome sensors NLRP3 and NLRP12, and its associated proteins ASC and pro-caspase-1 (Fig. 5A). The protein level of NLRP3 in prostate cancer cell lines was variable and corroborated with the expression pattern of mRNA and IHC. Although, we observed some variability in NLRP12 protein size at 95 kDa and 70 kDa, the constitutive level of NLRP12 was low to non-detectable in LNCaP and LNCaP-Ln3 cell lines as compared to aggressive prostate cancer cell lines, and corresponds with the expression profile in archival human prostate tissues. The level of inflammasome adaptor proteins ASC and pro-caspase-1 was variable across the prostate cancer cell lines. We observed a strong expression of an active form of ASC in PC3 cells while very low to non-detectable in other cancel cell lines. Since this antibody recognizes a splice variance, prostate cancer cell lines showed expression of lower size ASC form (~16 kDa). Additionally, we consistently observed a proteins size of about 30 kDa for ASC which could be an additional splice variance. The downstream targets, IL-1β and IL-18 pro-forms were primarily confined to aggressive prostate cancer cell lines PC3 and DU145 (Fig. 5A). While IL-18 pro-form is low to non-detectable in LNCaP, LN3, 22Rv1, and VCaP, a longer incubation also revealed a very low expression of IL-1β pro-form in prostate cancer cell lines. Although, inflammasome complex proteins were dominantly present in PC3 and DU145 cells, we did not find the mature/cleaved forms of IL-1β and IL-18. To examine the functional activity of IL-1β and IL-18 pro-forms, incubation of PC3 and DU145 protein cell lysates with human recombinant caspase-1 revealed the active and mature forms of IL-1β and IL-18 (Fig. 5B).

## Discussion

Analysis of differential gene expression has been rewarding in identifying new molecular targets associated with the growth and development of various cancer types. Current strategies to target prostate cancer are based on differential expression of prostate-associated genes (e.g. PSA; prostatic acid phospahatase (PAcP); prostate specific stem cell antigen (PSCA); androgen receptor (AR), etc.). However, expression profiles in association with inflammation regulatory pathway are limited despite the fact that inflammation plays a critical role in prostatic diseases^16, 17^. Previous studies reported that higher levels of inflammatory cytokines correlate with poor prostate cancer outcomes^18, 19^. Therefore, to better understand the regulation of inflammation, recent studies are attentive in exploring the role of inflammasome complex, which are exclusively limited to the cells of myeloid lineage^20, 21^.

Since inflammasomes regulate inflammatory cascade, the study of inflammasome complex proteins is fascinating to understand the role of inflammation in prostate cancer. Inflammasomes assembly regulating inflammatory state in the prostate has been shown in rat models where chronic prostatitis induced by intra-prostatic injection of carrageenan or benign prostatic hyperplasia (BPH) inflammation by formalin leads to NLRP1 activation and its downstream signaling IL-1β and IL-18 cytokines^22, 23^. Additionally, a melanoma study showed that inhibition of inflammasome and IL-1β suppresses the growth of tumor cells^24, 25^. We examined the expression of inflammasome complex proteins NLRP3, NLRP12, ASC, and pro-caspase-1, which are upstream of IL-1β and IL-18 signaling. NLRP3 expression was heterogeneous in stromal, benign and cancerous prostate tissues with no distinction between the adjacent benign vs. cancer tissues. However, other studies have shown an association of increased NLRP3 with the promotion and metastasis of gastric cancer, enhanced proliferation and migration of lung cancer cells, and colitis-associated colon cancer^26–28^. In support of our experimental observations, NLRP3 expression prolife in various prostate tissue types (no pathological alterations vs normal adjacent of tumor vs tumor tissues or metastatic prostate cancer) did not reveal any difference, suggesting no differential expression in association with prostate cancer progression.

Studies on the expression of ASC (apoptosis-associated speck-like protein containing a CARD) and caspase-1 with reference to inflammasome in cancer are limited. ASC serves as a bridge molecule between inflammasome receptors (NLRs) and pro-caspase-1. The observed level of ASC protein in different cell lines supports previous studies, which showed that ASC is downregulated due to hypermethylation in several cancer types including prostate cancer^29, 30^. Similar to our findings for low caspase-1 expression, it is shown that caspase-1 is weakly expressed in non-neoplastic and prostate tumor tissues with cytoplasmic localization^31, 32^. To perform regulated function of inflammasome activity, it is suggested that ASC translocate from nucleus to the cytosol and localizes with NLRs and pro-caspase-1^33^. We found that an active form of ASC is highly expressed in PC3 cells as compared to other cell lines indicating the functional activity of inflammasome complex in the cytoplasm during aggressive prostate cancer. However, understanding the role of different ASC forms in prostate cancer remains to be investigated.

The expression of NLRP12 was significantly higher in prostate cancer compared to adjacent benign tissues. It has been shown that overexpression of NLRP12 activates NF-κB and caspase-1, leading to IL-1β secretion in macrophages^34, 35^. Both, NF-κB and IL-1β has been associated with bone metastasis of prostate cancer^36^. Indeed, activation of NF-κB in human prostate carcinoma is also linked with biochemical relapse^37, 38^. Increased expression of NLRP12 in prostate cancer suggest that NLRP12 may play an important role in activating NF-κB and IL-1β signaling, and its association with the pathogenesis and progression of prostate cancer. Clinical studies have provided evidence that the measurement of IL-1β and IL-18 correlates with the risk of carcinoma and the prognosis of established cancer including prostate^39–42^. Despite strong expression of pro-forms of IL-1β and IL-18 proteins in PC3 and DU145 cells, the mature forms of IL-1β and IL-18 were not detectable in the culture supernatants (measured by ELISA: data not shown). However, digestion of cell lysates with recombinant caspase-1 showed mature forms of IL-1β and IL-18. It is likely that additional factors are required to activate and organize the formulation of NLRP12 inflammasome complex, similar to activation of AIM2 by the interferons (IFNs) in prostate cancer cell lines activating AIM2 inflammasome leading to interleukins IL-1β and IL-18 production^43^. Therefore, increased constitutive NLRP12 level in prostate cancer indicates that the NLRP12 may serve as a sensor of endogenous danger signals (e.g. uric acid and urinary carcinogens toxic to the prostate epithelial cells), regulating inflammatory cytokines creating inflamed environment stimulating prostate cancer cell growth. Identifying such factors leading to inflammasome activation and maturation of IL-1β and IL-18 will help in understanding the role of inflammation in the prostate tumor microenvironment.

To the best of our knowledge, this is the first study analyzing the sensor protein of inflammasomes complex and demonstrating the association of NLRP12 in prostate cancer. Since inflammasome assembly is considered a master regulator of inflammation, determining the molecular function of NLRP12 as an upstream regulator of NF-κB, IL-1β, and IL-18 signaling will add to our understanding the molecular basis of inflammation induced prostate cancer. Targeted inhibition of NLRP12 inflammasome may further help to appreciate the impact of inflammation on the biology of prostate tumors, and providing new directions investigating the role of inflammasomes in prostate cancer.

## Methods

### Reagents

Cell culture medium RPMI 1640 and serum free keratinocyte medium (SKM), phosphate buffer saline (PBS), fetal bovine serum, gentamicin, penicillin streptomycin, sodium pyruvate, L-glutamine, trypsin-EDTA, reverse transcriptase reagents, and SYBR green master mix was purchased from Invitrogen (Carlsbad, CA) through Fisher Scientific. Enhanced Chemiluminescence (ECL) reagent kit and the x-ray films were purchased from Mid Scientific (MO). Anti-NLRP3 and human recombinant caspase-1 from Millipore, anti-NLRP12 from GeneTex (Irvine, CA), anti-Caspase-1, β-actin, and horseradish peroxidase-conjugated secondary antibody was obtained from Cell Signaling Technology, Inc. (Danvers, MA). Antibodies to IL-1β, and IL-18 were obtained from Santa Cruz Biotechnology (Dallas, TX, USA), and ASC antibody was purchased from AdipoGen (San Diego, CA).

### Cell lines and cell culture

A panel of human prostate cancer cell lines (RWPE1, PzHPV7, LNCaP, 22Rv1, VCaP, PC3, and DU145) was purchased from American Type Culture Collection. LNCaP-Ln3, a derivative of LNCaP cells was obtained from Dr. Fidler (MD Anderson Cancer Center, TX). Cell lines LNCaP, Ln3, 22Rv1, VCaP, PC3 and DU145 were maintained in RPMI cell culture media containing 10% FBS, and other supplements as required. RWPE1 and PzHPV7 cells were maintained in SKM medium containing media supplements.

### Gene Expression Omnibus (GEO) data analysis

Gene expression datasets on prostate tumor tissues were retrieved using GEO profiles for various NLRs and AIM2 from the NIH website (http://www.ncbi.nlm.nih.gov/geoprofiles), and were analyzed for differential gene expression.

### RNA isolation and quantitative PCR (qPCR)

Total RNA from RWPE1, PzHPV7, LNCaP, LNCaP-Ln3, 22Rv1, VCaP, PC3, and DU145 cells was extracted using Qiagen RNA isolation kit following manufacturer’s instructions. RNA concentration was measured using BioTeK reader. Total RNA (~2 μg) was reverse transcribed using superscript II following standard methods. The cDNA concentration was measured, and 100 ng of cDNA was used for qPCR amplification using gene-specific primers and SYBR green master mix in StepOnePlus Real-Time PCR System (Applied Biosciences). Oligonucleotide primers for absent in melanoma (AIM2), NLRC4, NLRP3, NLRP6, and NLRP12 gene were synthesized from the previously published sequences using PrimerQuest Tool. GAPDH serves as internal control, and amplification in PzHPV7 cDNA was used to normalize the gene-specific expression.

### Immunohistochemistry (IHC)

De-identified archival specimens of prostate tumor biopsy were obtained from the Bio-specimen repository at the University of Kansas Cancer Center (Kansas City, KS), following approval from the institutional review committee, and all methods were performed in accordance with the relevant guidelines and regulations. Immunohistochemical (IHC) staining to analyze NRLP3 and NLRP12 protein expression was performed at the histopathology core facility of the University of Kansas Cancer Center. In these experiments, four micron sections of formalin-fixed paraffin-embedded tissue mounted on Epic Scientific Plus slides were air-dried overnight then baked for 60 minutes at 60 °C. Slides were deparaffinized and rehydrated in the traditional manner. Epitope retrieval was performed in a Biocare Decloaking Chamber (pressure cooker) for 5 min. using citrate buffer (pH 6.0). After the pressure returns to zero, slides were cooled to room temperature for 10 min., and then transferred to TBS Auto Wash (Biocare Medical, Concord, CA). Endogenous peroxidase was quenched with 3% H_2_O_2_ for 10 min. followed by addition of anti-NLRP3 or anti-NLRP12 primary antibody (Millepore, Temecula, CA), titer 1:300 for 30 min. A MACH2 Rabbit HRP-Polymer (Biocare Medical) detection reagent was applied, followed by a DAB chromogen for 5 min. Staining was performed using a Biocare Medical IntelliPATH automated stainer at room temperature. A light hematoxylin counterstain was applied and slides were dehydrated, cleared and mounted using permanent mounting media.

### Evaluation of Immunohistostaining

A certified pathologist (Dr. Ossama Tawfik at Kansas University Medical Center) with expertise in prostate pathology reviewed the slides for the intensity and extent of NLRP3 and NLRP12 staining blinded to the patient’s history. For NLRP3 analysis, we stained 20 samples, while for NLRP12, 50 specimen slides were stained, of which 40 contains matched benign, prostatic intraepithelial neoplasia (PIN), and malignant tissues on the same slide, and were chosen to compare the level of NLRP12. To allow for uniformity in scoring, each sample was given a composite score based on intensity and extent of tissue staining by the pathologist. Intensity score was graded on a four point scale corresponding to its numeric score: no staining of cells (0), weakly stained cells (1), moderate staining (2), and strongly stained cells (3). The extent of staining was graded on a four point scale as 1 (0–24%), 2 (25–49%), 3 (50–74%), and 4 (75–100%). The composite score was calculated by multiplying the values of intensity and extent of staining together. Clinical significance of the observed composite score was analyzed with respect to age, PSA and Gleason’s score.

### Western blot analysis

Human prostate cancer cells were seeded at a density of 5 × 10^5^ cells per well in a six-well plate. After 48 hours, cells were harvested, cell lysate were prepared in radio immunoprecipitation assay (RIPA) buffer containing fresh protease inhibitor mixture. Protein concentration was determined using BioTek reader and 30 μg of protein was loaded on to SDS-polyacrylamide gel and transferred electrophoretically to PVDF membrane (Bio-Rad). The membrane was blocked in 5% bovine serum albumin (BSA) in TBST (Tris-buffered saline containing 0.05% Tween 20) and incubated with appropriate primary antibody at a 1:1000 dilution. The membrane was washed in TBST and incubated with secondary IgG HRP conjugate at 1:5000 dilution, and the specific band size of the protein expression was visualized with ECL reagent exposed onto BioMax Film (Kodak).

### Statistical Analysis

The data was analyzed by Student’s t-test using GraphPad Prism software (GraphPad Software, Inc., CA). The value of P < 0.05 was considered statistically significant.
